# Experiences of cervical screening participation and non‐participation in women from minority ethnic populations in Scotland

**DOI:** 10.1111/hex.13287

**Published:** 2021-06-17

**Authors:** Mia Nelson, Andrea Patton, Katie Robb, David Weller, Aziz Sheikh, Kalpana Ragupathy, David Morrison, Christine Campbell

**Affiliations:** ^1^ Usher Institute The University of Edinburgh Edinburgh UK; ^2^ Institute of Health and Wellbeing University of Glasgow Glasgow UK; ^3^ Ninewells Hospital Dundee UK

**Keywords:** cancer screening, cervical screening, ethnicity, migrant populations, minority populations, qualitative comparison groups

## Abstract

**Background:**

The introduction of screening in the UK and other high‐income countries led to a significant decrease in the incidence of cervical cancer and increase in survival rates. Minority ethnic groups are often underrepresented in screening participation for reasons that are poorly understood.

**Objective:**

To explore experiences of cervical screening participation and non‐participation of women from minority ethnic populations in Scotland and gain insights to support the development of interventions that could potentially support screening participation and thereby reduce inequalities.

**Design:**

Qualitative comparison group study using in‐depth, semi‐structured individual interviews that were thematically analysed.

**Setting and participants:**

This study took place in Scotland. Fifty women were purposively sampled from four ethnic minority groups: South Asian; East European; Chinese; and Black African or Caribbean. White Scottish women were also interviewed.

**Results:**

Many experiences described were common regardless of ethnicity, such as difficulties managing competing priorities, including work and care responsibilities. However, important differences existed across the groups. These included going abroad for more frequent screening, delayed introduction to screening and not accessing primary care services, language difficulties in health‐care settings despite proficiency in English and not being sexually active at screening commencement. Experiences of racism, ignorance and feeling shamed were also reported.

**Conclusions:**

Key differences exist in the experience of minority ethnic groups in Scotland. These offer potential opportunities to reduce disparity and support screening participation including maximizing co‐incidental interactions and developing outreach work.

## 1 INTRODUCTION

Cervical cancer is a leading cause of morbidity and mortality in women, with an estimated 570 000 new cases and 311 000 deaths worldwide in 2018.^1^ However, cervical cancer is also one of the most successfully prevented and treated forms of cancer. Cervical screening aims to reduce cancer incidence through the early detection and treatment of precancerous changes and the introduction of screening in the UK and other high‐income countries in the late 1980s led to a significant decrease in the incidence of cervical cancer and increase in the survival rate.^2^, ^3^, ^4^, ^5^

However, the decrease in incidence in these countries has not been universal with minority ethnic groups underrepresented in screening uptake and over‐represented in cancer incidence compared with the majority White populations in some contexts.^6^, ^7^, ^8^, ^9^ In Canada, access of cancer screening is markedly lower among members of visible minority populations than the White population.^7^ Similarly, studies in the United States report lower access to cervical screening in minority populations along with a higher incidence of cervical cancers and increased mortality rates compared with women from the majority White population.^10^ While limited data exist for the UK as a whole, variation in cervical screening coverage by ethnicity has been reported at district level in England.^11^

While non‐participation sometimes results from informed decision making,^12^ studies including or focussing on minority populations suggest lower awareness of cervical screening^13^ and attitudinal and emotional barriers^14^ including fear, embarrassment, shame and absence of symptoms also contribute to the reduced uptake of screening among minority population in the UK. However, the relationship between non‐attendance and such barriers is complex with the same barriers also reported among individuals who do regularly attend screening.^15^, ^16^ Reducing health inequalities, particularly in cancer, is a priority in the UK with screening and early diagnosis key foci.^17^, ^18^ Previous studies exploring barriers in cervical screening have been conducted with individual minority and ethnically diverse populations; however, limited comparisons can be made across the studies to understand how experiences of minority populations differ due to contextual differences. The use of comparison groups within qualitative studies (eg groups with differing social positions such as patients, relatives and doctors,^19^ or different lived experiences such as people with or without a condition^20^) can facilitate identification of the group idiosyncrasies and phenomenological differences that impact the experience of health care, which often remain obscured or presumed inherent when using non‐comparative methods.^21^, ^22^ This study aimed to explore and compare the experience of cervical screening participation and non‐participation of women from different minority ethnic populations in Scotland, as well as White Scottish women, using qualitative comparison groups. We aimed to gain insights to support the development of interventions to reduce potential inequalities and support screening participation in minority ethnic populations in this region.

## 2 METHODS

### 2.1 Study design

This was an exploratory, descriptive study using qualitative comparison groups and individual, semi‐structured interviews with women from four ethnic minority groups that represent the largest ethnic minority populations in Scotland^23^: South Asian; East European; Chinese; and Black African or Caribbean. White Scottish women were also interviewed for comparison. Participants self‐defined their ethnicity and ethnic group.

### 2.2 Recruitment

Ten participants of each ethnicity were recruited through a specialist research recruitment company. Participants were recruited via an established database, social media and snowball sampling using a purposive sampling approach. Participants received £40 for their participation. The sample size was determined to ensure a variety of experiences within and across the groups.

Women were eligible if they were aged 30‐65 years (inclusive); currently resident in Scotland; and self‐identified as South Asian, East European, Chinese, Black African or Caribbean, or White Scottish. Participants were required to undertake the interview in English (with support of a friend/family if they wished). The exclusion criteria were as follows: informed consent not provided; previous or current diagnosis of cervical cancer; or ineligible for cervical screening (eg not having a cervix due to surgery or other reasons).

### 2.3 Procedure

Recruitment and data collection took place between August and October 2019. Interviews were conducted in English, took place face‐to‐face in the participants’ homes and were audio‐recorded. Participants lived around Edinburgh, Glasgow and Stirling, the most densely populated areas of Scotland. Interviews lasted between 30 and 100 minutes. Informed written consent was obtained prior to the interview. MN conducted all the interviews.

A topic guide, based on previous research in this area, was developed to facilitate participants’ recall and description of their experiences of screening (Appendix S2). Each interview followed its own course; the interviewer free to pursue any line of conversation appearing to be of importance to that participant. However, as we intended to compare experiences between groups, the topic guide was also used to ensure that all core topics (experience of participation; reasons for not participating; barriers/facilitators from their cultural perspective; views on acceptable approaches to women in their communities) were raised with every participant.

### 2.4 Data analysis

Interviews were transcribed verbatim using a professional service. Transcripts were read for accuracy by the interviewer (MN), pseudo‐anonymized and uploaded to a qualitative data management software (QSR NVivo12).

We undertook thematic analysis.^24^ Transcripts were first read for familiarity. Analysis involved a detailed examination of each transcript in turn, coding all the transcripts within one comparison group (defined by ethnicity) before moving on to the transcripts of the next group. As this study was designed with qualitative comparison groups, structural coding^21^ was employed first, with the topic guide used to segment the data in line with the core topics. An open coding approach was then used, identifying common topics and patterns within responses in individual transcripts. Codes were then clustered into themes and individual transcripts were examined in relation to others within the group, flagging any responses that were noticeably different within a group or theme. Once analysis was complete within each group, comparative analysis across the groups commenced.

MN conducted the analysis in full, and the research team (CC and AP) examined 30% of the transcripts (spread evenly over the comparison groups). The interpretations and thematic allocations made by MN were then discussed and reviewed during a series of team meetings over the course of the analytical process.

## 3 RESULTS

Fifty women were interviewed aged 30‐62 years (see Table 1 for demographic details). While each comparison group included participants with a range of demographic characteristics, none of the participants in the South Asian group and all of the East European group had migrated to the UK as adults. A full list of themes and subthemes with example quotes is provided in the supplementary material (Appendix S1).

**TABLE 1:** Participant demographics

	Black African or Caribbean n = 10	South Asian n = 10	Chinese n = 10	East European n = 10	White Scottish n = 10
Age (y)					
30‐39	4	3	6	8	5
40‐49	5	4	3	1	2
50‐59	1	3		1	2
60+			1		1
Marital status					
Single (never married)	4	2	2	5	3
Married	4	6	5	4	7
Separated	2	1	3		
Divorced		1		1	
Education					
Upper secondary education	1	1	1		
Post‐secondary non‐tertiary education	2	4	4	2	3
1st‐stage tertiary education (under‐grad)	3	4	2	5	4
2nd‐stage tertiary education (post‐grad)	4	1	3	3	3
First language					
Cantonese			2		
English	10	8	5		10
Mandarin			3		
Polish				9	
Punjabi		1			
Romanian				1	
Urdu		1			
Religion					
Agnostic					1
Buddhist	1		2		
Catholic	1			6	2
Christian	7		2	1	
Hindu	1	1			
Muslim		6		1	
Sikh		1			
Spiritualist		1			
No religion		1	6	2	7
Time in UK (y)					
5‐9			2	3	
10‐14	3			5	
15‐19	1			1	
20+	1		3	1	
Life	5	10	5		10

### 3.1 Experiences of participating in screening

Screening was universally reported as being unpleasant, but important, and many of the experiences described were common regardless of ethnicity. Similarities and differences in themes at the group level are demonstrated in Table 2.

**TABLE 2:** Experiences of participating in screening (● denotes presence of this theme in the group)

Section Code	Themes	Black African ‐ Caribbean	South Asian	Chinese	East European	White Scottish
Response to letter	Emotions – Fear, Anxiety, Dread	●	●	●	●	●
	Practical considerations	●	●	●	●	●
	Engage	●	●	●	●	●
	Avoid		●		●	
Getting an appointment	Easy enough	●	●	●	●	●
	Issues and difficulties – GP related	●	●	●	●	●
	Issues and difficulties – workplace related	●	●		●	●
	Issues and difficulties – general	●	●			●
	Wait a couple of weeks – viewed as good	●	●	●	●	●
	Wait a couple of weeks – viewed as bad	●	●	●	●	●
During screening	Neutral / Positive experiences		●	●	●	●
	Changing Experience	●		●		●
	Pain	●	●	●	●	●
	Practitioner's Gender	●	●	●	●	●
Talking about screening	That it is important	●	●	●	●	●
	Comparing experiences	●	●	●	●	●
	Up to a point	●	●			●
	To Understand	●		●	●	
Not talking about screening	We just don't talk about it	●	●	●	●	●
	Embarrassing—sexualized body part		●	●	●	●
	Cultural/Generational	●	●			
	Not with men	●				●

#### 3.1.1 Response to invitation letter

Many women across the groups described emotional responses to receiving the invitation letter relating to fear, dread or nervousness, recalling the physical or social discomfort experienced during previous examinations. Practical considerations were similarly common, including the need to plan round menstrual cycles, arrange childcare and/or time off work or wear clothes that would make the process simpler.

Dread, to start with. It's like, oh, goodness. Oh, God. Like so I’m quite prudish so just the thought of having to be in that physical position of having it done. (South Asian participant)

While those responses were frequent and common across the groups, a few participants described responses in keeping with avoidance, ranking screening as a low priority in their day‐to‐day life and perceiving themselves as low risk. These were only reported by women in the South Asian and East European groups but were unusual responses even within those groups reflecting individual differences rather than a common belief.

I was like, oh, I don't need it, I’m young, you know, I don't think it's necessary for me. Like I’m sure I’m going to have to check myself in a few years, not quite yet. (East European participant)

#### 3.1.2 Getting an appointment

Obtaining an appointment was typically considered quite simple because it was with the nurse, not the doctor. While flexible work hours, supportive work environments and extended GP opening hours were described as helpful by some participants, others cited limited work flexibility, working in male‐dominant environments and limited GP hours as problematic. Many participants commented that they needed to ‘wait a couple of weeks’ for an appointment. While some used this as an example of their difficulties in getting an appointment, just as many used it as an example of the ease with which they obtained one. These experiences were, again, common across the comparison groups.

It's always been within a reasonable timeframe, week, two weeks, to get in with the nurse and get it done. (South Asian participant)

Getting the appointment sometimes is very difficult because it takes two weeks. (Chinese participant)

#### 3.1.3 During the screening

Some participants, across all the groups except the Black African/Caribbean group, presented their experience of the examination itself in a neutral or positive way, often linked to the idea that it was quick. A few participants reflected on how their experience of the screening process had changed over the years. While one participant described becoming more self‐conscious as her body had aged, others discussed feeling more socially and physically comfortable with the process, often attributed to childbirth. Experiencing pain during screening was also common within and across the groups with participants describing it as *‘very uncomfortable’*, *‘always painful’* and *‘really, really sore’*.

There were mixed views within and across the groups about the health practitioner's gender. Many women discussed how they preferred female practitioners, and while some expressed this view as a relaxed preference, others were ardent about it. This was common across the groups. Some participants suggested that the practitioner's gender was unimportant; this was not, however, an experience expressed by any of the South Asian participants. Regardless of preference, the majority of participants across the groups assumed that the practitioner would be female and had only been screened by a female practitioner. Three participants discussed having had cervical screening performed by a man. These exceptions referred to screening undertaken 25‐35 years ago (White Scottish group) or abroad in a country where screening is routinely performed by gynaecologists (East European group).

#### 3.1.4 Talking and not talking about screening

There was a diverse range of experiences of both talking and not talking about cervical screening within and across the groups. Many participants across the groups believed that it was something that just was not talked about because the topic related to a sexualized body part and was embarrassing. Participants in the South Asian and Black African and Caribbean groups indicated that there were also broader cultural influences hindering conversations.

It's not something that you like…see the older generation, they would never talk about stuff like that anyway, kind of thing, I think. They were quite prudish, for want of a better word, about all these kind of things. (South Asian participant)

Participants suggested that there were some occasions where they did talk about screening including reminding female friends and family members that it was important to go, or to compare experiences or check something they were unsure about. However, limitations remained about the scope of the conversations and it was suggested that these were still not conversations to be had with men.

A few participants in the Black African and Caribbean, Chinese and East European groups reported having conversations with friends and family to obtain basic factual information and understand what cervical screening was after receiving their first invitation letter. These particular participants had migrated as adults and had not experienced screening before in their country of birth or in the UK.

I don't have any knowledge about it before. I don't know anything about it. You're seeing your name, date of birth…everything is correct. And cervical cancer screening…do I have a cancer? Oh my God, you know. It was, like…she is, like, my neighbor… I said, I don't know, I received a letter and said about cervical screening,… she said, oh no, no, that's…they do it for women every day, every three years. (Black African/Caribbean participant)

### 3.2 Experiences of not participating in screening

All the participants had attended cervical screening at some point in their lives and were still currently eligible. However, many women within and across the groups had experience of being out of date with their screening. Delays ranged from months to years and several participants were more than a year overdue for their screening at the time of the interview.

Four themes were identified in the participants’ experiences of delaying their cervical screening: competing demands; knowledge and risk perception; emotions; and system or process barriers. (Table 3).

**TABLE 3:** Experiences of not participating in screening (● denotes presence of this theme in the group)

Section	Theme	Subthemes	Black African–Caribbean	South Asian	Chinese	East European	White Scottish
Delayed screening	Competing demands	Looking after dependant	●	●	●		
		Competing health needs	●	●		●	
		Work	●	●	●	.	●
		Generally busy	●	●	●	●	●
	Knowledge and risk perception	Asymptomatic—why do it		●	●		
		It's screening not treatment				●	●
		Didn't realize importance				●	●
		Uncertain about what it was			●		
	Emotions	Embarrassment		●		●	●
		Pain	●	●		●	●
	System or process barriers	Couldn't get appointment			●		
		Problems making the phone call		●	●		
		Matching own/GP availability	●	●		●	
		Moving house / around country	●		●	●	●
Going in the end	Changing focus of fear	Importance overrides fear	●	●		●	●
	Persuasion from friends/family	Opportunistic approach by GP		●	●		
		Reminded by family/friend		●		●	
	GP/system reminders	Being chased up by practice	●	●	●		
		Reminder letters		●	●	●	

#### 3.2.1 Competing demands

Participants across the groups discussed problems managing competing demands on their time, energy and attention. The welfare of dependants and work‐related duties were discussed as being of greater importance than screening. This was portrayed not as a conscious decision to not screen, but as repeated short delays, where screening was perpetually planned but never top priority, or as an anticipated fixed‐term delay while waiting for a dependant's acute needs to ease or a busy period at work to pass.

I didn't put it in a priority. Yeah, normally, just you care about your children more than yourself. So, you just think, maybe not, yeah, you put something lower in your schedule, yeah. (Chinese participant)

Participants in the Black African/Caribbean, South Asian and East European groups described delaying their screening due to their own competing health needs. This most often related to being or trying to become pregnant, although there were also instances where significant acute illness had left individuals feeling less resilient and unwilling to undertake screening at that time.

As well as these specific identifiable issues, participants across the groups also talked of non‐specific competing demands with multiple pressures on their time and energy. Here, participants talked of simply forgetting or of deliberately delaying screening as a time management strategy, waiting to combine screening with other reasons for attending their GP practice.

And I remember delaying it, or not delaying it, just being so busy with everything else in life and putting it off. (Black African/Caribbean participant).

#### 3.2.2 Knowledge and risk perception

A small number of participants talked of delaying their screening as they believed their risks were low or viewed screening as a low priority generally, often linking this to the absence of symptoms. Although an identifiable theme within the study, it was unusual within each group and was not seen at all in the Black African/Caribbean group.

And then you think, I’m healthy, I will just, okay, I’ve got to get an appointment but not…it won't be that urgent. (Chinese participant)

#### 3.2.3 Emotions

Embarrassment and fear of the pain were themes that were, again, identifiable across the study but unusual in each group, and emotional experiences for delaying screening were not identified at all in the Chinese group. It is also of note that, while pain was a common theme when discussing the experience of screening, only a few participants attributed pain for their delay in obtaining screening.

The first‐time round they sent me the letter and I pretty much ignored it, I didn't want to go … it was painful, and it was difficult, and I was really embarrassed, and I didn't want to go back and go through that again. (Black African/Caribbean participant)

#### 3.2.4 System and process barriers

Women in Scotland receive an open invitation to screening via the post. Many participants across the groups, except for the White Scottish group, cited difficulties in making that appointment as a reason for their delayed screening including a lack of available appointments at the GPs, problems matching appointment availability with their own availability and difficulties in phoning to then book the appointment.

Other participants across the groups, except for participants in the South Asian group, discussed delays related to moving home. Participants had not received their screening invitation, and while some suggested this was because they had not informed their GP of their new address, others were unsure why their letters had not arrived. A few participants talked of not registering or deliberately not updating their details with the GP as they were on short‐term lets and moved out of their GP’s catchment area on multiple occasions.

So, since moving here, and moving to a new surgery, I thought I would get like a letter to remind me that it's approaching, and I know that it's probably due, or maybe even overdue, and I’ve not had any word about it. (Black African Caribbean participant)

### 3.3 Ending the delay

Participants who had experience of being out of date with their screening were asked to reflect on how their delay had ended. Four themes were identified: changing focus of fear; persuasion from friends and family; GP/system reminders; and community awareness. (Table 3).

#### 3.3.1 Changing focus of fear

Some participants who had expressed concern over anticipated pain and embarrassment described how their focus changed over time with the attention shifting from the potential pain to the potential consequences of not being screened.

They're the things that make me feel like, oh, it's not going to be nice, but, no, an awareness of the importance of having it done for definite overrides the feeling of, oh, I don't want to get it done. (South Asian participant)

#### 3.3.2 Persuasion from friends and family, and GP/system reminders

Reminder letters and phone calls from the surgery were often talked of as providing the impetus to stop delaying. However, some participants talked of requiring several reminders before they took action. As well as letters and phone reminders, a few participants had been approached by their GP while consulting on a different matter. Such reminders and direct approaches were viewed positively by the participants in this study.

How did she say it? You've not had your smear test … I went, okay. I said, oh, I’ll make it next time. No need to bother, I’ll do it now. (South Asian participant)

### 3.4 Key differences in experiences

While many screening experiences were broadly similar across the groups, there were a few key areas that differed. These areas included going abroad for screening while living in the UK, routes to screening and accessing primary health‐care services, language difficulties in health‐care settings despite proficiency in English and not being sexually active by the age of screening commencement.

In addition to these group differences, several participants discussed experiences that had been particularly difficult. Although the particulars of each participant's experience were different, they each illustrated experiences of marginalization. (Table 4).

**TABLE 4:** Key differences in experience (● denotes presence of this theme in the group)

Section	Theme	Subthemes	Black African ‐ Caribbean	South Asian	Chinese	East European	White Scottish
Screening elsewhere	Screening abroad while living in Scotland	More frequent timeframe				●	
		Accessing a specialist				●	
		General or female health check			●	●	
		Easier to communicate—Language			●		
	Starting UK screening/Registering with GP	Not registering with a GP	●		●	●	
		Pregnancy/Post‐natal check	●			●	
		Initiated by contraceptive need				●	
		Guided by partner / close friend	●			●	
		Directed by university			●	.	
Language	Difficulties	Difference medical–social English			●	●	
		Slang and accent	●				
		Telephone				●	
		Embarrassed to ask again	●			●	
Identified across multiple sections of the interview	Ignorance, racism and lack of representation	Experienced in Scottish NHS		●	●		
		Lack of representation in Scottish NHS	●				
	Feeling shamed	Weight					●
		Cutting	●				
	Not yet sexually active at 25 y old	Not going to be screened			●		
		Going, but not screened			●		
		Not being believed			●		

#### 3.4.1 Screening abroad while living in Scotland

Among the East European participants, Polish women discussed travelling to Poland to access specialist doctors, to have a broader health check including screening and to have screening on a yearly basis none of which was available in the UK. While some participants had spent many years only undertaking their screening in Poland, while living in Scotland, others described undertaking screening in both countries. All of the East European participants had migrated to Scotland as adults.

Chinese participants also described undertaking screening as part of a general health check when visiting China, with language barriers also suggested as a motivating factor in this group. It was noted that only women in the Chinese group who had migrated to the UK as adults talked of travelling abroad for screening.

In Poland it's usually once per year. So, then they check if everything is okay and do other tests, or just look inside and check if everything looks okay inside you. Well I never went here [Scotland] for years. So, I always went when I went to Poland. (East European participant)

#### 3.4.2 Routes to screening and accessing primary health‐care services

Many participants who migrated to the UK talked of not registering with a GP or accessing health care for several years after their arrival, despite their right to do so. Participants in the Black African/Caribbean group discussed being hesitant as they associated doctors with illness rather than preventative health care, and they were not ill. In these instances, the participants did not register with a GP until it was necessitated by illness or pregnancy, with screening introduced thereafter. Although accustomed to preventative health care, several participants in the East European group also talked of only registering with a GP when it was necessitated by illness, pregnancy or contraception.

When I got pregnant, then I had to go and register with a GP. …Because in Africa, you don't go to the hospital unless you are sick, you just have to treat yourself. … I’m like, oh…I go, how do I do it? She [close friend] said, no you have to register at the GP. I’ve been telling you to register, you said no. (Black African/Caribbean participant)

In both groups, participants who had migrated discussed learning about Scottish primary health‐care services through their social networks, with partners and friends who were either born here or had lived in Scotland longer than they had guiding them in how to register with a GP. Chinese participants also talked of being informed by their work or university.

Migrant participants across the groups also discussed experiencing practical barriers to registering with a GP and therefore to accessing screening. For some, this related to short‐term occupancy of rental accommodation and the likelihood of moving out of a surgery catchment area multiple times. For others, the need to register in person and during working hours, to provide evidence of migration status, acted as a barrier.

When I come over here, obviously I had to go and register with the doctor. However, on the beginning, I was renting a house every six months, so that was quite difficult to do, so we were changing all the time. And eventually, when I moved to where I was living for a longer period of time, then obviously I went to a GP, and I registered myself. (East European participant)

#### 3.4.3 Language difficulties in health‐care settings

Again, participants who had migrated to the UK described experiencing difficulties with language in health‐care setting despite being otherwise proficient in English. This also included participants in the Black African/Caribbean group who grew up in English speaking African countries. Difficulties arose around the difference between medical and social English, and the colloquial Scottish dialects and accents. These difficulties made some aspects of screening, such as phoning to make an appointment, more difficult.

I was very scared to phone, because I thought, what if I don't understand, and I felt embarrassed to ask three times, the same (East European participant)

#### 3.4.4 Not yet sexually active at 25 years old

Several Chinese participants talked about the fact that they were not yet sexually active when they received their first screening invitation. This experience was not identified in any of the other groups. While one participant had decided not to attend screening because of this, other participants had attended for their screening. The experience of those participants varied significantly. Although both identified their virginity to the practitioners, while one participant was advised to wait until she was sexually active the other participant described how she had been screened regardless, which left her feeling anxious and tense about subsequent screenings.

My first experience was I was at uni and I went to see the doctor and then she gave me a cervical screening…. I told her I wasn't sexually active or anything but she still went and did the screening, but I was quite sore, it wasn't something I anticipated at all…because of that soreness from the first… every time I go, I tense up, you know…I don't think the doctor believed until they did the smear and then she saw the blood. (Chinese participant)

#### 3.4.5 Marginalization

A number of particularly difficult experiences were raised by participants across the groups, with the exception of the East European group. Participants in the Chinese, South Asian and Black African/Caribbean groups talked of difficulties related to ignorance, racism and the lack of representation in the training and experience of health‐care practitioners. The experiences described related to both clinical and non‐clinical staff, and while some experiences were described as *‘not racist, just ignorant’,* others were experienced as racism.

I kind of realised that when I came to this GP here that you're not…they've never had any Asians before. He was surprised that I spoke English. … Just the comments that were made were I found quite demeaning. …But it wasn't…I would not…definitely not racist, just ignorant. (South Asian participant)

The receptionists at that practice were really horrible. That's why I changed GP. …One of them wouldn't talk to me when I approached them and went to the other receptionist ‘you need to deal with her.’ I was really, really down. …I think there was a racial thing going on there. Because it wasn't just me, it was any coloured person that went in. (Chinese participant)

Participants in the Black African/Caribbean group talked of a lack of representation in terms of a sparsity of health‐care practitioners in Scotland from minority ethnic populations and in terms of practitioners’ understanding of normal and abnormal physiology in Black and minority populations.

In addition, a small number of participants in the Black African/Caribbean group and the White Scottish group described experiences that left them feeling shamed by practitioners due to their physical difference. These differences related to cutting (female genital mutilation) and body size. While some of the difficulties related to thoughtless comments and inappropriately timed conversations, others involved looks, facial expressions and poorly masked reactions. The participant discussing body size spoke of her own experience. However, the participant discussing cutting had not been cut herself and was discussing experiences shared by older women in her social network in Scotland.

They catch you in the cervical smear, and you're saying, here I am, everything is exposed, and you start talking about my weight. … You know, I mean, the whole thing it becomes unbelievably excruciating, talk about it when I’ve got high blood pressure testing, that's fine, that is an appropriate…not when my legs are in the air. (White Scottish participant)

Being from Sierra Leone, one of the things, a stigma, is FGM. So, I’ve been in the room when other women, aunties, have been speaking about their experiences of giving birth, going for cervical screening, and receiving the reactions from nurses, that, ‘Oh my God, what's happened there?’ and the shame that goes with it. (African/Caribbean participant)

## 4 DISCUSSION AND CONCLUSION

This study examined experiences of participating and not participating in cervical screening in four minority ethnic populations and the majority ethnic population in Scotland. While many of the experiences participants discussed were common regardless of ethnicity, some key differences exist and offer insights to support the development of interventions to increase participation in screening among minority ethnic populations.

Pain and embarrassment were experiences seen across the groups in the anticipation of screening and in the recall of previous experiences of cervical screening and have been reported as potential barriers in other research studies with minority populations and in the general population.^25^, ^26^, ^27^ However, they were rarely cited by participants in any group in this study as an actual barrier when reflecting on previous episodes of delayed cervical screening.

Competing demands and practical factors, in comparison, were commonly reported as barriers across the groups when reflecting on episodes of delayed screening. Practical barriers have been reported in ethnically diverse populations in England^13^ with work pressure, childcare commitments and limited GP opening hours being common themes. Our results additionally illustrated ways in which the postal system may act as a potential practical barrier to screening uptake in Scotland, with participants discussing deliberately and accidentally failing to update their postal address with their GP practice. Invitations have been identified as an effective method of improving uptake to cervical screening^28^ and reminders from the practice, by letter, phone and in person, were experienced by participants in our study as important factors in returning to cervical screening after a delay. A recent Scottish study explored the acceptability of different forms of communication for cervical screening^29^ and the results suggested that broadening the options in communication methods beyond letters may help increase screening participation in Scotland. However, although such measures may help increase screening across the population as a whole, it might not address inequity of access for minority ethnic populations.

Previous studies in the UK and other European countries have indicated a lower awareness of screening programmes and lack of understanding of the benefits of screening among ethnic minority populations in comparison to the majority population.^13^, ^27^ However, research within Black African populations in the UK and USA has suggested that limited awareness among the recent migrants plays an important role in shaping the lower uptake in this population.^30^, ^31^ Risk perception and limited awareness of screening were unusual themes within each of our groups; however, it was the participants who had migrated as adults, especially from regions where screening programmes are not routinely offered, who discussed being unaware of screening prior to the receipt of their first invitation letter.

It is also of note that many participants who had migrated did not register with a GP for several years after arriving in the UK, despite eligibility to do so. Cervical screening is predominately provided through GP services but is available to individuals not registered with a GP through sexual health and family planning services. Although this may be of limited benefit to a population with little awareness of screening, several of our participants indicated that their impetus to register, and their first screening opportunity arose through contraceptive need, pregnancy or postnatal care. These results illustrate the value of maximizing co‐incidental interactions to promote screening. Recent studies and reports in England and abroad, focussing on a range of health conditions as well as cervical screening, have demonstrated the feasibility and effectiveness of community‐based outreach programmes in addressing inequalities and reaching underserved populations.^32^, ^33^, ^34^, ^35^, ^36^ Strengthening efforts to engage with minority populations in the community, and in particular the migrant populations within them, may help address disparity in cervical screening participation in Scotland.

East European participants discussed travelling to Poland to access specialists for their screening, either instead or as well as screening in Scotland. This pattern has also been reported in England^37^ and noted in relation to breast cancer screening in Scotland.^38^ However, there were still instances where participants were not accessing screening in either country and it should not be assumed that non‐attenders are obtaining health care elsewhere.

Participants reported experiences of racism, ignorance and feeling shamed. Previous research undertaken in the United States,^39^, ^40^ New Zealand^41^ and Europe^42^, ^43^ has indicated that the experience of racial and religious discrimination in health‐care settings not only impacts trust and satisfaction with the health‐care services, but also acts as a barrier to accessing preventative care and leads to delayed help‐seeking. The experience of enacted/felt stigma in relation to bodyweight^44^, ^45^ and cutting^46^, ^47^ has similarly been found to negatively impact health‐care use and help‐seeking in the United States and UK. Addressing stigmatizing beliefs and practices among health‐care professionals and supporting the development of culturally sensitive and knowledgeable practitioners may help to promote cervical screening participation in minority ethnic groups in Scotland.

### 4.1 Strengths and limitations

We employed qualitative comparison groups. Although much work has been carried out with individual minority populations, limited comparisons can be made across the studies due to contextual differences. To our knowledge, this is the first study to qualitatively compare cervical screening experiences across groups of women from different ethnicities in the same contextual setting.

We intended to obtain a mix of demographic characteristics within each group. However, all the participants in the South Asian group grew up in the UK. While we did achieve a mix of demographics overall and elucidated some of the experiences particular to individuals who migrated as adults, experiences of South Asian migrants have not been captured in this study. Our sample was purposive and we did not attempt to represent any particular screening groups (eg non‐attenders/attenders). It is possible that those who participated in the interviews experienced fewer barriers to cervical screening and that both shared and ethnic group‐specific problems were less common than in the wider population. For example, all participants spoke English. However, although the participant's current language ability enabled them to participate fully, this had not always been the case and participants were able to reflect on past experiences and describe the ways in which language had impacted their experience of screening.

One researcher undertook all the interviews in this study. While there are arguments for having multiple interviewers in some study designs, qualitative comparison group methodology requires minimal variation between groups beyond their focal difference.^21^ Having one interviewer was, therefore, a strength in this particular study.

### 4.2 Conclusions

While many of the experiences reported were common regardless of ethnicity, key differences exist and offer potential opportunities to reduce disparity of access, including using alternative ways of identifying and communicating with women eligible for screening, maximizing co‐incidental interactions with individuals from minority and migrant populations, and developing outreach work with populations not otherwise accessing health care. While we found many examples of positive supportive practice, there is also an on‐going need to address stigmatizing beliefs and practices in health‐care staff and support the development of culturally sensitive and knowledgeable practitioners to support screening in Scotland.

## CONFLICT OF INTERESTS

All authors declare no conflict of interest.

## AUTHOR CONTRIBUTIONS

Mia Nelson contributed to research design, data collection, data analysis, interpretation of results and writing of paper. Andrea Patton contributed to data analysis, interpretation and writing of paper. Katie Robb, David Weller, Aziz Sheikh, Kalpana Ragupathy and David Morrison contributed to research design, interpretation of results and writing of paper. Christine Campbell contributed to research design, data analysis, interpretation and writing of paper.

## PATIENT OR PUBLIC CONTRIBUTION

Patient and public contribution is planned for this research in the incorporation and translation of the findings into practice and policy. The patient and public involvement activity is anticipated for 2021 having been delayed from Autumn 2020 due to the COVID pandemic.

## RESEARCH ETHICS

Edinburgh University's Research Ethics Committee approval was obtained in August 2019 (study number: 1922 – Usher Institute).

## Supporting information

Appendix S1

Appendix S2

## Data Availability

Research data are not shared.
