# Supplementary material for: Experiences of cervical screening participation and non‐participation in women from minority ethnic populations in Scotland
Source: Health Expect. 2021 Jun 17;24(4):1459–72. doi: 10.1111/hex.13287 (PMC8369098; doi:10.1111/hex.13287)
Supplement: Supplementary file 1 — Appendix S1 [file HEX-24-1459-s002.docx]

## Supplementary material – additional illustrative quotes for themes

**Experiences of participating in screening**

| **Section Code** | **Themes** | **Illustrative quotes** |
| --- | --- | --- |
| Response to letter | Emotions – Fear, Anxiety, Dread | Dread, to start with. It’s like, oh, goodness. Oh, god. Like so I’m quite prudish so just the thought of having to be in that physical position of having it done is...and then the build-up of this imaginary, oh my god, it’s going to be agony, and it’s never been agony. It’s uncomfortable for a few seconds, but it’s not agony. (South Asian participant) |
|  | Practical considerations | Oh, not again. And then it means that I have to wear a skirt, 'cause I don't want to wear trousers. Because trousers are harder to remove than a skirt, a skirt, you can just remove your underwear, and then that’s you sorted. (Black African/Caribbean participant) |
|  | Engage | I just think it's routine, I'm like oh okay, book the doctors and get the smear test done. Just get it out the road. (Chinese participant) |
|  | Avoid | It’s not a priority, I’ll be honest. And that sounds really bad but it’s like, oh, right, okay, I need to make an appointment, I need to go through and get it done, and obviously because it’s an invasive procedure, I think…well, I personally I tend to put it off so I have been quite bad in the sense that I usually end up waiting for a reminder to come through. (South Asian participant) |
| Getting an appointment | Easy enough | I just booked an appointment with the nurse as opposed to the doctor.  So, it was fairly easy and fast from what I remember, because the nurses have quite a lot of appointments. (South Asian participant) |
|  | Issues and difficulties – GP related | it’s pretty bad considering how much they scare you into needing to get it done and when you try and book an appointment, they’re like we’re not taking an appointments actually until next month now, we’ve closed our books. (Chinese participant) |
|  | Issues and difficulties – workplace related | My job is very much deadline, like deadline is an event so within certain times, especially like when the first letters came through, I probably had an event on.  So then for the next month I was super-busy in the lead-up to it, so there was no way for me to take time away off work. (East European participant) |
|  | Issues and difficulties – general | You can either get one within a week or sometimes it’s within three weeks or four weeks that you can get something that suits, kind of thing. I have on occasion like I’ve said, many a time that I’ve realised, oh, my cervical smear, oh, goodness I’m on my period so I’ll have to cancel it, and then once you’ve cancelled it you’re looking again. (South Asian participant) |
|  | Wait a couple of weeks – viewed as good | It was definitely easy. I just went to the reception and they checked the dates and it was pretty easy to get in. So I got it within the next two weeks. (East European participant) |
|  | Wait a couple of weeks – viewed as bad | In Edinburgh, it's a bit tricky. So if I call my surgery, it usually takes about two weeks, so it's quite tricky. (East European participant) |
| During screening | Neutral / Positive experiences | I don’t feel any way either way about it really. It’s just something, as I said, something that you have to do. It’s fine. (Chinese participant) |
|  | Changing Experience | When I was really young, I remember there was something and I was quite…I wasn’t bothered about it at all. I was quite comfortable. There was like five people at one point having a look. I think they were doing some kind of training where they had people and I was just a bit like…but now I wouldn’t feel that comfortable. I would never be comfortable with that now. … I’ve put on loads of weight and I’m a bit more flabby and that doesn’t look like…my vagina doesn’t look like the way it used to look. (Black African/Caribbean participant) |
|  | Pain | Someone has to look at your private parts and even worse it’s sore as well and I couldn’t get [relaxed], … so then it’s taking longer and the nurse has to try a few times. It’s very unpleasant. s(East European participant) |

Continued…

|  | Practitioner’s Gender | I've not had any guys. I think if it was a guy, I'd feel a bit awkward. …if I saw a guy I don't think I'd feel comfortable, I'd say I need to get a female, you'd just feel a bit awkward. …But then if I don't have a choice, then I'd be like okay, fine, but I think preferably I don't want a guy looking down there, preferably more a woman. (Prefer female/not to have male HCP – Chinese participant)  No, I’d refuse. … I don’t know, I don’t know, I would feel uncomfortable. I don’t really want anyone looking down there, who’s not a lady, I have no idea why. (Prefer female HCP – White Scottish participant)  I would prefer to have the female, and I would prefer to have the doctor who's seen everything, just because she's very thorough. But I don't really mind a male, because it's his job. (Doesn’t really matter - Black African/Caribbean participant)  My gynaecologist was a male, which again, I didn’t mind, because that’s what we always had. We were always being told that the male is more gentle than the woman. that was kind of, I don't know, a stigma, maybe not even a stigma, but that’s what we'd been told, you go to a male, it's much more gentle, and it's better. The woman can be not as gentle, and can be, not as nice. So we always went to a male, and obviously, it was kind of embarrassing at the beginning. (Prefer male HCP – East European participant) |
| --- | --- | --- |
| Talking about screening | That it is important | However, me, as me, I go, because I know it's important, and it's preventable. And even though sometimes I share on Facebook when it's, obviously, I think it's in June, the awareness. So I just usually send, even share this important, and even sometimes, I giving trouble to my friends if they don't want to go, I say, you have to go, that’s it, you have to go. (East European participant) |
|  | Comparing experiences | I was worried about, I’ve seen some blood on the sheet whenever she took out the tools, even though I wasn’t on my period. So that’s always quite worrying. I don’t think that I’ve ever asked about it, but I...yes, I spoke to my sister about it. And she said she had that as well. (East European participant) |
|  | Up to a point | You know, I wouldn’t just text them and go, oh, have you been for your smear?  It would be a conversation.  What have you been up to today?  Oh, well, I had to go for my smear.  I’d go, ouch, how was that?  Okay, fine and then we’d move on. (South Asian participant) |
|  | To Understand | I don't have any knowledge about it before. I don't know anything about it. You’re seeing your name, date of birth…everything is correct. And cervical cancer screening…do I have a cancer? Oh my God, you know. It was, like…she is, like, my neighbour… I said, I don't know, I received a letter and said about cervical screening, ... she said, oh no, no, that’s…they do it for women every day, every three years that…just to, you know, check if there any abnormalities and everything. (Black African/Caribbean participant) |
| Not talking about screening | We just don't talk about it | Oh, my god. Never, we never talk about it. No, you just presume, because I’m going for mine and checking that I’m alright, that everyone else is doing it, you never think to ask. Wow, that’s an eye opener. (Black African/Caribbean participant) |
|  | Embarrassing - sexualised body part | I think it’s that whole talking about your reproductive organs and, you know, that’s a bit embarrassing. (South Asian participant) |
|  | Cultural/Generational | It’s not something that you like…see the older generation, they would never talk about stuff like that anyway, kind of thing, I think. They were quite prudish, for want of a better word, about all these kind of things. (South Asian participant) |
|  | Not with men | No, I wouldn’t openly talk about it. But equally, if I'm going for a smear test, I don't find that embarrassing to say, I'm going for a smear test. So I might not say it to a male boss, for example, but like, I wouldn’t have any qualms saying to my mum or to sort of female colleagues at work, I've got my smear, kind of, a bit like that. (White Scottish participant) |

**Experiences of not participating in screening**

| **Section** | **Theme** | **Sub-themes** | Illustrative quotes |
| --- | --- | --- | --- |
| Delayed screening | Competing demands | Looking after dependant | I didn’t put it in a priority. Yeah, normally, just you care about your children more than yourself. So, you just think, maybe not, yeah, you put something lower in your schedule, yeah. (Chinese participant) |
|  |  | Competing health needs | I hadn’t been well myself, so sometimes I just, and I think, okay, I'll just give it a miss, and go another time…. I had taken septicaemia. So I was in hospital with that. And then when I came back out of hospital, it was like, it took a lot out of me so I was really kind of drained out, so I just couldn’t cope with having the smear test. (South Asian participant) |
|  |  | Work | Because with [company name] I worked away a lot of the time for a… It was a different company so I could be away for four months at a time, so I think that’s probably the only times but it’s not intentional, like I’ve delayed it. I just haven’t been able to get there. (White Scottish) |
|  |  | Generally busy | And I remember delaying it, or not delaying it, just being so busy with everything else in life and putting it off and then eventually going and then being really relieved that it was fine. (Black African/Caribbean participant) |
|  | Knowledge and risk perception | Asymptomatic - why do it | And then you think, I’m healthy, I will just, okay, I’ve got to get an appointment but not…it won’t be that urgent. (Chinese participant) |
|  |  | It’s screening not treatment | I know that if lots of things like that is left to my devices, it's going to be postponed, unless it is something really, you know.  You can argue, because it is important, it is very important, but because it is screening, it is not treatment, you feel like, oh okay, it doesn’t matter. (East European participant) |
|  |  | Didn't realise importance | I was in the army and it was never something that, sort of, crossed my mind.  It wasn’t really out there as, like, an urgent thing you had to get done, or anything.  It was only really since having my son and the Jade Goody thing that, sort of, made me more aware sort of thing. (White Scottish) |
|  |  | Uncertain about what it was | So I think I got about three letters by the time I went. I got that letter through after I registered with the GP and I didn’t want to go for it. Because I wasn’t sure what it was at that point, to be honest…I was postponing going for a long time. But I think one of the conversations with my sister when she got a letter as well, she was like, no, you need to go, you need to check yourself, it’s important. So then I started to go. (East European participant) |
|  | Emotions | Embarrassment | I would get the letter and I was delaying that as long as possible… I think, first of all, it’s shame and, well, you have to get naked there. Someone has to look at your private parts and even worse it’s sore as well.  (East European participant) |
|  |  | Pain | The first-time round they sent me the letter and I pretty much ignored it, I didn’t want to go … it was painful, and it was difficult, and I was really embarrassed, and I didn’t want to go back and go through that again. (Black African/Caribbean participant) |
|  | System or process barriers | Couldn't get appointment | Last time I phoned for an appointment. I phoned up, I got through, I wanted to get an appointment, I couldn’t book it, they had it on their diaries, and then I was on holiday for a month. So, by the time I finally got booked in it was probably three months after I got the letter. (Chinese participant) |
|  |  | Problems making the phone call | For my practice anyway I have to phone up at eight o’clock in the morning and even if I’ve been on the phone at the dot of eight o’clock or even a minute beforehand it will either be engaged or you just…I kid you not, you know when you keep pressing the number, it can be up to 30 times before you’ve got through. (South Asian participant) |
|  |  | Matching own/GP availability | I put it off a little bit but I do get it. Well there is just, yeah childcare and then booking time off work, so it is just whatever day that I can get off work. I work until half five, the doctors is only open until six, … you have to take a flexi half day. (South Asian participant) |
|  |  | Moving house / around country | So, since moving here, and moving to a new surgery, I thought I would get like a letter to remind me that it’s approaching, and I know that it’s probably due, or maybe even overdue, and I’ve not had any word about it. (Black African Caribbean participant) |
| Going in the end | Changing focus of fear | Importance overrides fear | They’re the things that make me feel like, oh, it’s not going to be nice, but, no, an awareness of the importance of having it done for definite overrides the feeling of, oh, I don’t want to get it done. (South Asian participant) |
|  | Persuasion from friends/family | Opportunistic approach by GP | How did she say it? You’ve not had your smear test or something and I went, okay. I said, oh, I’ll make it next time. No need to bother, I’ll do it now. (South Asian participant) |
|  |  | Reminded by family/friend | It was actually my husband who prompted me because you know when you have that list to do or papers et cetera.  He was going through them and he was like, you’ve still not made that appointment, have you?  I was like, oh, no.  He was like, you’d better make it, and I’m like, yes, I know, I know, I know. (South Asian participant) |
|  | GP/system reminders | Being chased up by practice | The first time round they sent me the letter and I pretty much ignored it, I didn’t want to and the practice manager knows our family pretty well, … and she was like, yeah [Name], you haven’t responded – 'cause I don’t want to go, last time it was really difficult’. … And I didn’t want to go back and go through that again. They were like, well why didn’t you say, you can tell us. … So they did the right thing in chasing me up and I went there …and I was really lucky because, yeah I would have happily ignored that phone call or the letters and just put my head down. (Black African Caribbean participant) |
|  |  | Reminder letters | I think, another letter, I think it said, I'm overdue, and I need to register. So I go, I just come to the GP. The appointment system was easy, so you'd go early in the morning, I think, and book on the day. And then I did it, and then, yeah, it's okay. (Chinese participant) |

**Key differences in experience**

| **Section** | **Theme** | **Sub-themes** | **Illustrative quotes** |
| --- | --- | --- | --- |
| Screening elsewhere | Screening abroad while living in Scotland | More frequent timeframe | In Poland it's usually once per year. So smearing is once per year but you can go more often. So, then they check if everything is okay and do other tests, or just look inside and check if everything looks okay inside you. Well I never went here [Scotland] for years. So, I always went when I went to Poland. Yes. So, when I went on holiday every year, I tried to go to the gynaecology and go there for the check-up. And then yeah, this… The first time here, in this year, in April this year, so I decided like oh, I'm living here, I need to start going here. (East European participant) |
|  |  | Accessing a specialist | When I go to Poland, I always go check myself, anyway. So, it's like kind of, extra, even though I'm attending the smear test here, but when I've got opportunity to go to Poland, I always make a point of the gynaecologist. I pay my share, what I have to pay, but I always check with the smear test, and I also check my breasts, and ovaries, everything, so I've got a scan. And this is kind of like, that’s what we do. (East European participant) |
|  |  | General or female health check | NHS is my main doctor here. I can't really go back China every time, every year, I can't afford it. So, I normally maybe go every maybe three years, so that's the time I go for my whole health check. (Chinese participant) |
|  |  | Easier to communicate - Language | Well, my mum did…she’s got health problems so she does go privately and all that sort of thing, she goes to Hong Kong for private screening and all that sort of thing. Yeah, and sometimes…she had one here for the BUPA, sort of thing but…’cause she can’t really communicate so she’ll do it in Hong Kong when she’s there for a visit. (Chinese participant) |
|  | Starting UK screening/Registering with GP | Not registering with a GP | When I come over here, obviously I had to go and register with the doctor.  However, on the beginning, I was renting a house every six months, so that was quite difficult to do, so we were changing all the time. And eventually, when I moved to where I was living for a longer period of time, then obviously I went to a GP, and I registered myself.  (East European participant) |
|  |  | Pregnancy/Post-natal check | Well when I came here, I came with my first born. I had her in Nigeria. And the second one, I think…when I got pregnant, then I had to go and register with a GP. Because I’m like, oh I missed my period and I don't know, maybe stress of being in another country, ‘cause of that. Because in Africa, you don't go to the hospital unless you are sick, you just have to treat yourself. When you know that it is getting worse, that’s when you go to the hospital. So when I noticed that, oh this is unlike me, maybe it’s the weather, this and that, then…so it’s now over a month…two months, I’m like, oh…I go, how do I do it? She [close friend] said, no you have to register at the GP. I’ve been telling you to register, you said no. (Black African Caribbean participant) |
|  |  | Initiated by contraceptive need | I came to Glasgow, first of all. And I needed contraceptive pills, so I think this was the first time I went to a GP to sign up. And I think, just the letters started coming. So I didn’t know about it, but the letters would usually inform me. (East European participant) |
|  |  | Guided by partner / close friend | Well, we were going to travel and my husband, when I first came, he said, oh, you have to register with a GP to get your shots to go travelling. …He is British … so he told me what to do because I would not have known what you have to do and all that so he said, oh, you register. … When it [THE INVITATION LETTER] first came, I went on the internet as well and looked at it so I knew, I had an idea what it was because I know over here there is so much more they do, like the screenings and all of that. You get all that and you get these letters sent out. I thought, well, I’d better go and do it. (Black African Caribbean participant) |
|  |  | Directed by university | I was registered with a GP automatically when I started studying here but at that time, yes, you’re young, you don’t really care and yes you don’t think it’s necessary to have a GP, you have one but you just don’t need it. (Chinese participant) |
| Language | Difficulties | Difference medical - social English | At that time, my vocabulary was not very enough, especially for the kind of special medical terms.  Sometimes you don't get what they're for.  But generally, I can understand most of the words.  (Chinese participant) |
|  |  | Slang and accent | It’s just sometimes they would speak fast and I couldn’t understand like some of the accents but now I can. (Black African Caribbean participant) |
|  |  | Telephone | I think I went over, I didn’t, I was very scared to phone, because I thought, what if I don't understand, and I felt embarrassed to ask three times, the same. So I used to go, and actually was face to face, and I prefer actually going and saying, right, that I would like to make an appointment. But then, obviously, when my English became better, then I was more brave to actually speak on the phone. (East European participant) |
|  |  | Embarrassed to ask again | Yeah, it's very difficult, because I knew…I think I was a little bit embarrassed as well, that I had to ask twice. And sometimes when I had to ask three times, I just nod. And then, obviously, my friend and my husband, they knew I didn’t have a clue, that I was nodding because I didn’t want to be rude. (East European participant) |
| *Identified across multiple sections of the interview* | Ignorance, racism and lack of representation | Experienced in Scottish NHS | The receptionists at that practice were really horrible. That’s why I changed GP. …One of them wouldn’t talk to me when I approached them and went to the other receptionist “you need to deal with her.” And I wasn’t doing anything. I was really, really down. To the point where I actually went into the GP and I burst out crying. …I think there was a racial thing going on there. Because it wasn’t just me, it was any coloured person that went in. But the doctors were lovely. The nurses were lovely. But reception... And I observed it when I was sitting there waiting for the doctor to come out. “You can’t do that”, you know, that’s the way she would talk to some people. Then a non-coloured person would go up and it was all, “hello, how are you, blah, blah, yes, thank you”. But then to a coloured person [tutting], oh. (Chinese participant) |
|  |  | Lack of representation in Scottish NHS | A lot of the African women that I work with, or used to work with, they would have preferred to have an African doctor, or whatever. But those are not the options that you have with the NHS in Scotland, you know. You might have come across one, but highly unlikely, and even less likely with a female doctor, you know. In fact, I don't think I've ever had a woman of colour as a nurse. No, I haven’t, I definitely haven’t. (Black African Caribbean participant) |
|  | Feeling shamed | Weight | With being large you always hit the, kind of, oh you’re making a fuss ‘cause you’re large, that sort of…It’s automatic, so people have an automatic thing against large people. Particularly in the medical profession, you know, first of all it’s, you know, you’re in with the flu and they’ll go, and what about your weight, and you feel like saying, I don’t give an f’ing stuff about my weight. I’ve got…I am ill. You know, this is not the right moment to catch me, actually. Or they catch you in the cervical smear, and you’re saying, here I am, everything is exposed, and you start talking about my weight. … You know, I mean, the whole thing it becomes unbelievably excruciating, talk about it when I’ve got high blood pressure testing, that’s fine, that is an appropriate…not when my legs are in the air. And I might be a nice jolly friendly person, but I also have feelings. (White Scottish participant) |
|  |  | Cutting | Being from Sierra Leone, one of the things, a stigma, is FGM, you know, genital mutilation. And thankfully my mum never took me to Freetown to do anything like that, so I’m really, really happy for that.. So, I’ve been in the room when other women, aunties, have been speaking about their experiences of giving birth, going for cervical screening, and receiving the reactions from nurses, that, oh my God, what’s happened there? That type of thing. And the shame that goes with it. … One thing that remains quite clear was about the reaction and the face of the person when you spread your legs. I think that also needs to be really worked into and just understood, …, like hearing it from individuals’ own mouths as to how that face, that stare, the questions, or maybe even how they were treated afterwards, makes them feel. (Black African Caribbean participant) |
|  | Not yet sexually active at 25 years old | Not going to be screened | Some people said, some people said, it's not good, if you are still a virgin. They said, it's just, it will hurt you, or it will break the film. So that’s why, that’s the only, I think that’s the reason why I didn’t go for it in the beginning.  I thought, I haven’t had any experience, so I'm at little risk I have a problem with this.  So, I think, I'll just avoid until maybe I'm more mature. (Chinese participant) |
|  |  | Going, but not screened | I just booked an appointment with a nurse, with my local GP in London, my first job was in London and then I went there and then before nobody told me you had to be sexually active, so I didn’t know that. It was just, okay over 25, I’ll just go to it and then the nurse told me you had to be sexually active, to do that. So, then I didn’t do it when I was just 25 and then I met my husband in London as well and after a while, I became sexually active. So, I went to do the screen. (Chinese participant) |
|  |  | Not being believed | My first experience was I was at uni and I went to see the doctor and then she gave me a cervical screening. And at that time, I told her I wasn’t sexually active or anything but she still went and did the screening, but I was quite sore, it wasn’t something I anticipated at all.…because of that soreness from the first… every time I go, I tense up, you know, I just have to relax or something, you know. … I don’t think the doctor believed until they did the smear and then she saw the blood and everything and then she realised that she…just because university students are sexually active, but I wasn’t, yeah. (Chinese participant) |
