# Supplementary material for: Experiences of cervical screening participation and non‐participation in women from minority ethnic populations in Scotland
Source: Health Expect. 2021 Jun 17;24(4):1459–72. doi: 10.1111/hex.13287 (PMC8369098; doi:10.1111/hex.13287)
Supplement: Supplementary file 2 — Appendix S2 [file HEX-24-1459-s001.docx]

**SCREEN Scottish Cervical Screening and Ethnicity**

**INTERVIEW GUIDE**

# *Check if there are any outstanding questions, ensure consent form is signed, demographics sheet is completed, and recorder is on.*

**Knowledge of cervical cancer and cervical screening**

- Is cancer of the cervix a type of cancer you’ve heard much / know much about? probe…
- What about cervical screening? Do the women in your family or social circle speak about it? probe…
- What different names/ terms used for cervical screening in your community?

**Experience of participation or reasons for not participating as appropriate**

- Do you remember receiving an invitation for cervical screening? (will have examples of invitation letters to act as trigger <http://www.healthscotland.com/documents/24327.aspx> )
- As best you can remember, when invitation letter arrived in the post what did you think and how did you feel?
- How did you decide what to do next? (e.g. Knew right away what you’d do/thought it over/don’t know/remember)
- Did other things happening in life at the time influence decision? Was this time different from other times? Is this how you have reacted in the past? How did you find it? (probes .. who did the smear, nurse or GP, how well explained, any concerns)
- If not, please tell me why not? What influenced your decision not to take part?
- Ask about if have attended screening in another country (e.g. for eastern European women in Poland), and ask about experience there, and any differences they have seen.
- Have you taken in either breast screening or colorectal screening either here or in any country? If you, ask about any differences they’ve seen.

**Barriers and facilitators from their cultural or religious perspective**

- In your community, what sorts of things might put people off having cervical screening?
- Are there differing views among younger and older women?

**Views on acceptable approaches to women in their communities in relation to cervical screening**

- Ask about proximity of their general practice, whether language is an issue…
- Are there other health facilities you would be willing to go to for a smear?
- Community support / other women being available…

**General understanding of HPV, HPV vaccination and cervical cancer**

- What do you understand about HPV? and HPV vaccination?
- What do you understand about how HPV and the vaccine are related to cervical cancer?
- Ask about HPV self-sampling
  - In the future, it may be possible for women to do the cervical screening test themselves at home, using a vaginal swab (similar to a cotton bud). Would this be of interest to you? why or why not?

**Closing**

- Are there any other issues about cancer in general or about cancer screening that we’ve not covered that you would like to raise?

*Thank participant for taking part; ask about results summary; ensure recorder is off, etc.*

**Snowballing**

You are welcome to pass on the email/Facebook message you received about the study to anyone (women) you think might be interested in taking part. Or they can contact TM directly to find out about this study and see if they are eligible to take part.
